# Convalescent Homes

**Published:** 1905-03-11

**Authors:** 


### CONVALESCENT HOMES.

### A NEW DEPARTURE.

At a meeting held on Thursday, 2nd inst., at 20 Hanover Square, under the chairmanship of Sir Edmund Hay Currie, it was unanimously resolved to form a "Convalescent Homes Association," consisting of convalescent institutions supplying charitable relief to London patients, the object being to form a representative council to centralise effort and to promote efficiency and economy.

The Chairman was supported by Sir William Church, president of the Royal College of Physicians; Mr. John Tweedy, president of the Royal College of Surgeons; Mr. Thomas Bryant, president of the Hospitals Association; Dr. Kingston Fowler, Dean of the Faculty of Medicine of the University of London; Lord Lytton, Lady Frederick Cavendish, delegates from the great hospital funds, and others, a number of superintendents of and others interested in convalescent homes being present. Letters of regret were read from Sir William Broadbent, Sir Victor Horsley, Sir Thomas Barlow, Mr. George Herring, Sir Savile Crossley, and others unable to be present.

The chairman congratulated Dr. Habershon and Mr. Fitzgerald, at whose instance the meeting was convened, on the success of their initial effort to concentrate public opinion on the question. Recent discussion in the press indicated a growing feeling that some of the homes did not entirely meet present day needs, and the object of the meeting was to hear the opinions of the medical profession on the one hand and proprietors of homes on the other. From intimate knowledge of the subject he held that such an association as was proposed was a necessity. He wished, however, to enter an emphatic protest against the creation of another general fund, and the building of a new Home. Either, in his opinion, would be fatal to the future usefulness of the existing homes no less than to the great London hospitals, which had shown themselves for many years alive to the necessity of "cure homes" for their patients. It was on the understanding that the promoters of the association were in agreement with him on this point that he was able to occupy the chair and to give his support to the scheme.

#### Homes of Rest.

Sir William Church, in proposing that the increasing needs of medicine and surgery require some alteration in the character of relief afforded by convalescent homes to the poor of London, preferred the phrase "Homes of Rest," some of these institutions having lost sight of their original use as secondary hospitals, or places of recovery after illness. People merely requiring change of air and rest should be dealt with by some such organisation as that which boarded out children in the country or at the seaside. Mr. Tweedy, in seconding, referred to improved methods of surgery which made it possible to pass an enormous number of patients through the wards, thus making largely increased space at convalescent homes an absolute necessity.

Sir Thomas Smith, Sir Douglas Powell, and Col. Montefiore (representing the Charity Organisation Society), having supported the resolution, Mr. Fry, on behalf of King Edward's Hospital Fund, referred to the waste in connection with those homes which were too full in the summer and empty in the winter. The fund which he represented was glad to promote anything which would lead to the better treatment of the suffering poor, and he cordially supported the resolution, which was carried unanimously.

It was then unanimously agreed that an association be formed on the lines indicated above, the council to consist of one representative from each convalescent institution and from each of the great general hospitals of London, and of such other lay and medical representatives whom it might be considered advisable to invite, the Presidents of the Royal College of Physicians and Surgeons, the Dean of the Faculty of Medicine of the University of London, the Chairman of the Central Hospital Council, and one representative from each of the great hospital funds to be *ex officio* members.

Sir Henry Burdett, K.C.B., in proposing that a small executive council be elected from the General Council, added an amendment to the effect that Mr. Fitzgerald, chairman of the largest convalescent institution in the metropolis, should be asked to serve, and that Dr. Habershon, to whose energy and interest it was due that the meeting had been called together, should be invited to act as honorary secretary. It appeared to him that there was a great deal of work to be done in connection with the movement, and that suitable persons would have to be found to do it, and with two such gentlemen in office the association would have the assurance that the executive would be strengthened and the work well done. He made this proposal because he regarded the meeting as among the most important departures in matters of medical reform that had taken place for many years past, and because he felt that it was most important that the whole of the bed accommodation in hospitals and the convalescent institutions should be brought into line and utilised to the best purpose. There was no doubt that the position of isolation which these institutions had occupied in the past had led to many anomalies, and he looked forward to this meeting and the work which would result from it as giving to the subscribers a guarantee that every convalescent institution in this country which was doing good work would be affiliated, they in their turn, by affiliation, giving a guarantee of thoroughly good work. If his amendment were accepted he ventured to think that day's enterprise would be set on foot under the best possible auspices. He believed success was possible, and that it could best be secured in the adoption of the proposal he had made. Dr. Habershon having signified his willingness to accept office, the meeting terminated with a vote of thanks to the chairman, and to the Medical and Chirurgical Society for the use of the room.

